# VODCA: Verification of Diagnosis Using CAM-Based Approach for Explainable Process Monitoring †

**DOI:** 10.3390/s20236858

**Published:** 2020-11-30

**Authors:** Cheolhwan Oh, Jongpil Jeong

**Affiliations:** Department of Smart Factory Convergence, Sungkyunkwan University, 2066 Seobu-ro, Jangan-gu, Suwon 16419, Korea; dhdldzhkf13@skku.edu

**Keywords:** class activation map, deep neural network, statistical process control, fault detection and diagnosis, anomaly detection

## Abstract

Process monitoring at industrial sites contributes to system stability by detecting and diagnosing unexpected changes in a system. Today, as the infrastructure of industrial sites is advancing because of the development of communication technology, vast amounts of data are generated, and the importance of a way to effectively monitor such data in order to diagnose a system is increasing daily. Because a method based on a deep neural network can effectively extract information from a large amount of data, methods have been proposed to monitor processes using such networks to detect system faults and abnormalities. Neural-network-based process monitoring is effective in detecting faults, but has difficulty in diagnosing because of the limitations of the black-box model. Therefore, in this paper we propose a process-monitoring framework that can detect and diagnose faults. The proposed method uses a class activation map that results from diagnosis of faults and abnormalities, and verifies the diagnosis by post-processing the class activation map. This improves the detection of faults and abnormalities and generates a class activation map that provides a more verified diagnosis to the end user. In order to evaluate the performance of the proposed method, we did a simulation using publicly available industrial motor datasets. In addition, after establishing a system that can apply the proposed method to actual manufacturing companies that produce sapphire nozzles, we carried out a case study on whether fault detection and diagnosis were possible.

## 1. Introduction

With recent advances in the industry, the use of machinery has diversified to the point at which it can be found anywhere in life, and with the proliferation of the internet of things, it is possible to collect data that could not have been previously collected from the existing systems. Accordingly, the need for a technique to effectively prevent faults in a system by effectively managing the collected data has increased.

In various industries, statistical process-control (SPC) technology is effective in improving the process by means of fault detection and diagnosis (FDD). However, problems, such as multicollinearity and false alarms, occur when high correlation and nonlinearity exist in the data [1]. Therefore, large-scale, complex industrial processes require appropriate monitoring techniques to process large-scale data efficiently. Thus, studies have been proposed that combine the traditional SPC method with machine learning and deep-learning techniques that effectively analyze large datasets [2].

The purpose of machine learning in process monitoring is to learn latent patterns present in training data and then to recognize patterns in future data for which no prior information is known. In this case, the performance of a machine-learning model that uses the extracted features as training data depends greatly on how the training data’s latent pattern is extracted. Extracting latent patterns from training data is called feature engineering and is considered to be an essential process in machine learning [3]. If feature engineering is not done correctly, it is challenging to guarantee performance even if more advanced machine-learning models are used. Conversely, when the latent patterns of data are effectively extracted by means of feature engineering, high performance can be achieved even with a relatively simple machine-learning model. However, feature engineering is the most labor-intensive process in machine-learning pipelines, and even after the pipeline is designed, it has a difficulty in that it must be applied differently depending on the domain to which it is applied.

Unlike machine learning, a deep-learning model automatically extracts and learns latent patterns that exist in the training data by itself. The deep-learning model is built up by stacking multiple hidden layers, which automatically extract features from the data [4]. For the advantages above, studies incorporating deep learning in the field of process monitoring that must deal with raw data occurring in real time are attracting attention [5].

However, deep-learning methods are considered black-box models and cannot explain the internal calculation mechanism. In other words, even if the fault is accurately detected by the deep-learning model, it is difficult to diagnose why this fault occurred. The difficulty of explaining the decisions made by deep-learning models is a problem that arises not only in manufacturing applications but also in almost all fields that want to use deep-learning models.

To solve this problem, researchers have proposed various methods for eXplainable Artificial Intelligence (XAI). Among them, representative methods are the Class Activation Map (CAM) [6] proposed in the convolutional neural network (CNN)-based architecture and attention mechanism [7]. By means of these methods, studies to explain the decisions made by deep-learning models are being conducted in various applications, and studies for fault diagnosis are also being proposed in manufacturing applications [8].

However, the XAI methods do not always give accurate results and often give completely wrong results. In [9], which proposed a visualization method using the XAI method, there are examples of mistakes that can occur when explaining the decisions made by a deep-learning model. Therefore, a causal analysis of deep-learning models using XAI must always be carried out carefully, always keeping in mind that incorrect analysis results may occur. This requires even more caution in process monitoring, which can lead to cascading damage in continuous processes because of incorrect diagnoses.

In this paper we propose an explainable neural-network-based process-monitoring framework that can detect and diagnose system faults. The framework we propose diagnoses faults based on CAM. For verification of a diagnosis, we do post-processing on the CAM used to diagnose faults. The post-processing is done according to garbage in, garbage out (GIGO). It works by naming the CAM that occurs when the FDD process fails as garbage CAM and treating it as an outlier.

The rest of this paper is organized as follows. Section 2 discusses the related work. Section 3 describes a control chart for performing process monitoring. Section 4 describes the proposed method. Section 5 presents the experiments we did to evaluate the proposed approach’s performance and describes the experimental result. Section 6 discusses conclusions and future work.

## 2. Related Work

In the manufacturing process, accident prevention and product-defect reduction are among the most important goals. Thus, it is important to detect and diagnose machine faults. For decades, FDD has been actively researched. First, fault detection evaluates whether the process operates under normal circumstances. Then, fault diagnosis finds out the characteristics and cause of the fault.

The FDD method can be broadly classified into two categories: model-based and data-based methods. The model-based approach develops and applies process models in fault detection. The method is very reliable and has been widely researched, developed, and applied in process systems. However, it is difficult to capture system complexity in nonlinear models, and early fault detection in complex processes cannot be easily implemented [10].

In contrast, data-driven methods are measured based on minimal process knowledge. Data-driven FDD methods can be classified as qualitative or quantitative. The expert system and qualitative trend analysis are representative qualitative techniques [11].

Quantitative methods can be categorized as statistical or non-statistical. As a statistical method, process monitoring based on SPC sets a control limit based on data collected from a stable state [12]. Support vector machines and artificial neural networks are the most commonly used non-statistical supervised learning methods, and both construct a learning model of recorded data with labels and do FDD by comparing the model with the current process data. The downside of this FDD technique is that it requires numerous labeled data samples for training [13].

In particular, because process monitoring using a neural network is a black-box model, it is difficult to interpret the relationship between input data and output results in the actual manufacturing process despite high classification performance [14]. Accordingly, the problem arises that end-users in a system with FDD based on neural networks must rely on models that can make mistakes. In this situation, the end-user may lose trust [15].

To improve this, research has been conducted to overcome the limitations of the neural network’s black-box model and to propose fault diagnosis by means of an explainable artificial-intelligence model [16]. Among them, the CAM, which can be applied to CNN-based deep-learning models, is effective in mitigating the black-box problem [6].

The CAM is a method to provide the model with explanatory power by visualizing which part of the image the CNN model predicted. The CAM uses the weight of the target class in the last convolutional layer to highlight an important region as a localization map when predicting a class in an image. However, the CAM has the restriction that the global average pooling (GAP) layer rather than the fully connected (FC) layer must be enforced in the last layer. The recently proposed Grad-CAM improved this using the gradient of the target class in the last convolutional layer [17]. Accordingly, the Grad-CAM can be used in CNN models, such as VGG with FCN. Moreover, the CAM and Grad-CAM have been used in several fields to improve the explanatory power of deep-learning models. In addition, in FDD, the CAM or Grad-CAM can be used for a visual explanation [8].

## 3. Control Chart in Process Monitoring

Rather than monitoring the process with a guess, SPC finds solutions or improvements by identifying and interpreting problems based on data. It is primarily used for quality control [12]. In general, the control chart technique is divided into univariate or multivariate control charts depending on the number of data features to be monitored.

### 3.1. Univariate Control Charts

The control chart has been used as an essential tool in statistical process control since it was first proposed by Shewhart of Bell Labs in the United States in 1931 [18]. Control charts have been widely used to manage production processes statistically and are an effective method for estimating process parameters and analyzing process capability. The Shewhart chart is the most widely used chart, because it is efficient and straightforward to use. It has the advantage of being accessible and easy to apply, but because it judges the state of the process using only the current observation values, it is inefficient in being less sensitive to small or slow changes in the process.

The cumulative sum (CUSUM) control chart increases the efficiency of the control chart by using both past and current observation values to judge the status of the process [19]. The exponential weighted moving average (EWMA) chart is a widely used with the Shewhart chart and the CUSUM chart [20]. EWMA control charts are weighted and averaged not only for current data but also for past data; so they have the advantage of being sensitive to small changes in the process and are widely because easily used.

### 3.2. Multivariate Control Charts

When a univariate control chart is used for process monitoring, it has the advantage of being simple to use and intuitive to analyze, because data is used directly without going through a mathematical model. However, in cases where this is applied to multivariate data, a problem arises, in that the correlations between multiple variables cannot be considered. Also, using a univariate control chart for each variable is inefficient for the end user [21]. If it is necessary to manage multivariate variables simultaneously, multivariate control charts that can be monitored by integrating them into one can be useful. To this end, various multivariate control charts have been proposed, such as the multivariate CUSUM chart, the multivariate EWMA chart, and Hotelling’s $T^{2}$ chart [21], which is the most widely used [22]. The $T^{2}$ statistic is calculated using Equation (1):

(1) $$T^{2}={\left(x-\bar{x}\right)}^{T}S^{-1}\left(x-\bar{x}\right),$$

The $T^{2}$ statistic represents the mahalanobis distance, which can reflect the covariance between multivariate variables. Here $\bar{x}$ and *S* correspond to the sample mean vector and covariance matrix of historical data $X\in R^{n\times m}$, and *n* and *m* are the numbers of data samples and variables, respectively. The historical data *X* is collected in the control state. Moreover, based on this, the upper control limit (UCL) of the $T^{2}$ statistic is set. By means of this, process monitoring is done by checking whether the $T^{2}$ statistic calculated from the newly collected data follows the distribution of the data collected in the control state. Given the assumption of normality of the data, the $T^{2}$ chart can set the upper control limit according to the *F*-distribution as follows [12]:

(2) $$UCL_{T^{2}}=\frac{m(n+1)(n-1)}{n(n-m)}F_{\left(m,n-m,\alpha \right)},$$

In Equation (2), the α, significance level, is the type 1 error rate. This is a threshold for the maximum amount of errors that incorrectly judge a fault as normal. Moreover, $F_{\left(m,n-m,\alpha \right)}$ is the upper α th quantile of the *F*-distribution with *m* and (n−m) degrees of freedom.

However, the process monitoring using a Hotelling’s $T^{2}$ chart is not useful for data with numerous correlated variables. If there are many such variables, inverting the covariance matrix *S* is challenging because the covariance matrix becomes almost singular, which leads to problematic results [23]. In addition, when the data includes many highly correlated variables, these may cause multicollinearity; thus, the ability to detect a progress shift may deteriorate [1]. Therefore, instead of using raw data, a control chart based on latent variables has been proposed to overcome the problems above by means of proper preprocessing.

### 3.3. Multivariate Control Charts Based on Latent Variables

Hotelling’s $T^{2}$ chart, which uses principal component analysis (PCA) to extract latent variables, is a representative multivariate control chart based on latent variables. PCA is an unsupervised learning technique used for dimensional reduction, visualization, and clustering. This technique uses an orthogonal transform to identify principal components, which equal a linear combination and have no linear correlation with each other [24]. This is similar to the $T^{2}$ chart without PCA described in Equations (1) and (2), but the main difference is that in order to do residual analysis, the *Q* chart is also applied to process monitoring. Residual analysis is done on the latter of the principal component subspace (PCS) and residual subspace (RS), which are decomposed by means of PCA. RS is a part that cannot be reflected by the number of selected principal components *p*. Therefore, the result of residual analysis depends on how many *p* are selected.

(3) $$X=\hat{X}+\tilde{X}=\hat{T}{\hat{P}}^{T}+\tilde{T}{\tilde{P}}^{T}=\left[\hat{T}\;\tilde{T}\right]{\left[\hat{P}\;\tilde{P}\right]}^{T}=TP^{T},$$

where $T=\left[\hat{T}\;\tilde{T}\right]$ and $P=\left[\hat{P}\;\tilde{P}\right]$ are the score and loading matrices. $\hat{T}\in R^{n\times p}$ and $\tilde{T}\in R^{n\times m-p}$ are the score matrices that belong to PCS and RS, and $\hat{P}\in R^{m\times p}$ and $\tilde{P}\in R^{m\times m-p}$ are the loading matrices that belong to PCS and RS. When *p*, which is the hyperparameter to do PCA, is selected, the $T_{PCA}^{2}$ statistics are calculated by means of Equations (4) and (5).

(4) $$\hat{t}=x\hat{P},$$

(5) $$T_{PCA}^{2}=\hat{t}{\hat{\Lambda }}^{-1}{\hat{t}}^{T},$$

where $\hat{t}$ is the score vector of *x* in the PCS and $\hat{\Lambda }$ is the diagonal matrix of the largest eigenvalues of the covariance matrix of $\hat{X}$. The upper control limit of the $T_{PCA}^{2}$ is obtained by means of Equation (6), which is similar to Equation (2) described above.

(6) $$UCL_{T_{PCA}^{2}}=\frac{p(n+1)(n-1)}{n(n-p)}F_{\left(p,n-p,\alpha \right)},$$

However, because the $T_{PCA}^{2}$ statistic obtained by means of Equation (5) reflects only the information corresponding to the PCS, the concern exists that variations in the RS cannot be detected [25]. Thus, the $Q_{PCA}$ statistic is also used to detect shifts that cannot be reflected by only the information corresponding to the PCS. The $Q_{PCA}$ statistics can be calculated from residuals obtained from RS. The $Q_{PCA}$ chart monitors the squared error between the vector $\hat{x}$ estimated by means of PCA and the true vector *x*. The $Q_{PCA}$ statistic is obtained by means of Equation (7):

(7) $$Q_{PCA}=\left(x-\hat{x}\right){\left(x-\hat{x}\right)}^{T}=\tilde{x}{\tilde{x}}^{T},$$

Assuming the normality of the $Q_{PCA}$ statistic obtained by means of Equation (7), the $UCL_{Q_{PCA}}$ can be set through an approximation calculated based on a weighted chi-square distribution and is obtained by means of Equation (8) [26].

(8) $$UCL_{Q_{PCA}}=\frac{v}{2m}\chi_{\left(2m^{2}/v,\alpha \right)}^{2},$$

In Equation (8), the α means the type 1 error rate, as mentioned above, and *m* and *v* correspond to the sample mean and variance of $Q_{PCA}$, respectively. Moreover, this still works well even if it violates the assumption of the normality of the $Q_{PCA}$ statistics [27].

A multivariate control chart using latent variables extracted by means of PCA has the advantage of easing the multicollinearity problem between multivariate variables. However, it does not well reflect the nonlinearity of the data with the PCA-based latent variables extracted by means of linear combination.

To address this, several methods to reflect nonlinearity have been proposed in prior studies; among them, the kernel method is one of the most widely used techniques. The kernel method reflects nonlinearity by mapping raw data to a high dimension. Kernel methods owe their name to the use of kernel functions, which enable them to operate in a high-dimensional, implicit feature space without ever computing the coordinates of the data in that space, but rather by simply computing the inner products between the images of all pairs of data in the feature space. This operation is often computationally cheaper than the explicit computation of the coordinates. This approach is called the kernel trick [28].

In [29], Kernel PCA (KPCA) was proposed to combine the kernel method with PCA. KPCA is applied in many domains, because it can reflect the non-linearity of data and is easy to use. In the process-monitoring domain using multivariate control charts, studies using KPCA have been proposed and showed that monitoring performance could be improved [30,31].

Recently, studies have been proposed that apply deep neural networks as a method for extracting latent variables. In [2], a method of extracting latent variables using a variational autoencoder (VAE), an unsupervised deep neural network model, was proposed and then applied to a multivariate control chart. The researchers showed that the latent variables extracted by means of VAE can mitigate nonnormality and nonlinearity problems.

## 4. Proposed Method

This section describes Verification Of Diagnosis using a CAM-based Approach (VODCA), which is the FDD framework we propose. VODCA has the purpose of providing reliable fault diagnosis results while applying neural-network-based process monitoring to the manufacturing site. The proposed framework follows a three-step process:

- Fault detection by CNN
- Fault diagnosis by CAM
- Process monitoring based on VAE

In the first process of our proposed framework, the collected industrial data are analyzed using a neural-network-based classifier to detect whether the current system has reached a fault condition. After neural-network-based fault detection is done, a second process is used to diagnose the data classified as a fault in the system by generating CAM. Since we use CAM as a diagnosis result for a fault, we use CNN-based architecture as a neural-network classifier for fault detection to generate CAM. The CAM explains the causal relationship between model inputs and outputs through the feature map’s weight information. Through this process, the temporal pattern of the data causing the fault is diagnosed.

In the final process-monitoring step, the end user is provided with a dashboard to monitor the FDD results. This monitoring dashboard does VAE-based process monitoring to prevent misdiagnosis and provide easy diagnosis results. The proposed process monitoring is done according to the GIGO logic.

In computer science, GIGO logic is the concept that nonsense input data produces nonsense output, regardless of the data processing method. In this paper, the GIGO logic works similarly to anomaly detection based on unsupervised learning using an autoencoder that learns only normal data. In the case of inputting abnormal data to an autoencoder trained using only normal data, the autoencoder cannot properly reconstruct the abnormal data because it has never learned about the abnormal data. In other words, a significant difference occurs between input data and output data, and when the difference exceeds a certain level, it is treated as an outlier [32]. Here, abnormal data can be called garbage data. The difference between the above-described method and our proposed method is that normal data and abnormal data are directly generated through the class activation mapping.

Previous studies that proposed a method of detecting faults in production facilities using a deep neural network-based classifier are often performed up to the first step of the proposed framework in this paper. These studies focus on improving the performance of the classifier. However, in this study, we admit that even a well-designed classifier cannot always detect a fault with 100% accuracy. Accordingly, we focus on monitoring whether misclassification has occurred during the fault detection step.

Whereas GIGO-based process monitoring assumes that the CAM corresponding to a well-predicted true positive (TP) and true negative (TN) in the preceding fault detection has similar manifolds, the CAM corresponding to the incorrectly predicted false positive (FP) and false negative (FN) exhibits anomalies. This process starts from the point at which the predicted label data, which are the output of the fault detection model, are needed to calculate the CAM. Initially, when fault detection of the CNN model is done incorrectly, the predicted label data input to calculate the CAM becomes garbage; thus, the output CAM is also assumed to be garbage data.

Process monitoring is performed by training the VAE model, which learns the manifolds of CAM only corresponding to the TP and TN produced in the fault detection and diagnosis process. After the VAE model is trained, the latent variable *z* containing the manifold of the CAM corresponding to the TP and TN is calculated using the encoder of the VAE to set the upper control limit $UCL_{T_{PCA}^{2}}$ and $UCL_{Q_{PCA}}$ of the PCA-based $T_{PCA}^{2}$ and $Q_{PCA}$ charts introduced in Section 3. The *z* extracted by means of the VAE model is robust in the $T_{PCA}^{2}$ and $Q_{PCA}$ charts based on the normality assumption because they follow a normal distribution [2]. Through PCA, latent variables that do not have a linear correlation with each other are identified. Finally, in the test phase, the CAM generated by means of the misclassification and misdiagnosis that may occur in FDD is subjected to post-processing by means of VAE-based $T_{PCA}^{2}$ and $Q_{PCA}$ charts to do process monitoring. Through PCA, latent variables that do not have a linear correlation with each other are identified.

In other words, the control chart implemented as a latent variable *z* of the VAE model that learned only the manifolds of the CAM corresponding to the TP and TN does not judge this as an outlier when receiving the CAM generated with well-classified label information. Conversely, when the CAM generated with misclassified label information is received, it is treated as an anomaly.

If the last step of process monitoring is not done, because the reliability of the diagnosis results has not been verified, an end user may have doubts about whether the diagnostic results provided in the form of the CAM can be trusted. However, in the proposed method, VAE-based process monitoring can effectively identify the CAM that is considered garbage, so that end users can trust the diagnosis results provided to them.

**Figure 1:**
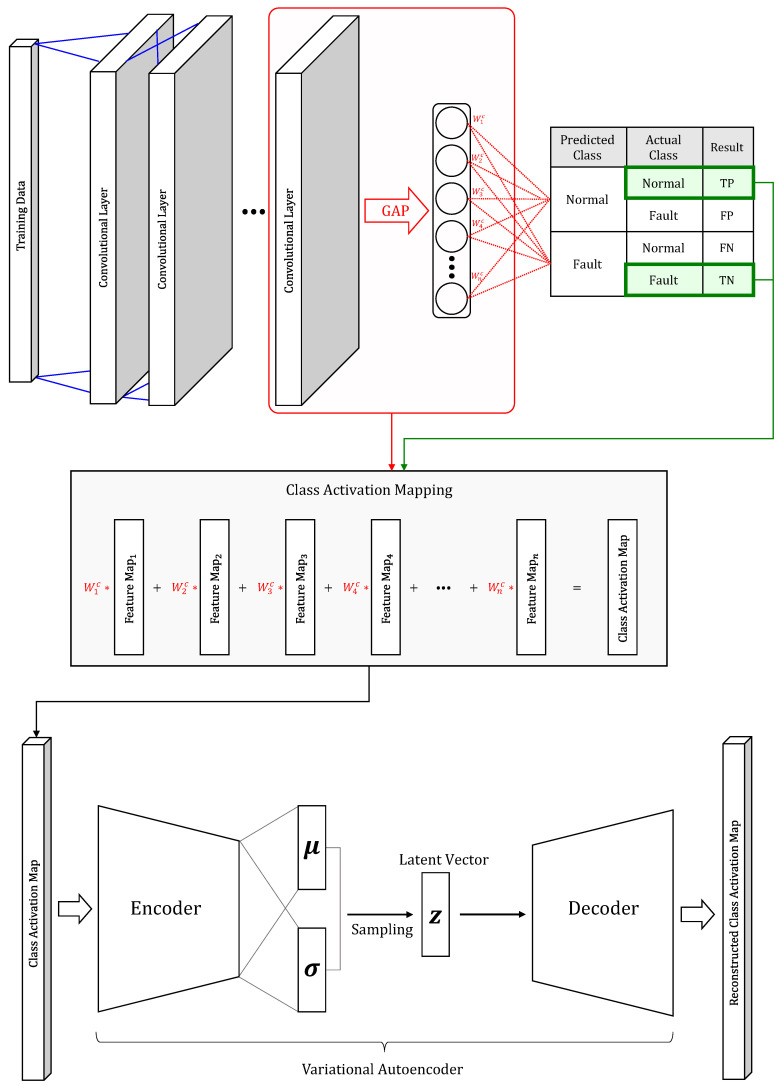
The training phase of the proposed method. VAE is trained by generating CAM only with training data corresponding to TP and TN.

Figure 1 illustrates the model training phase to perform the proposed method. Among the training data used in the learning process, the CAM is generated using only data classified as TP and TN. That is, the VAE is learned using only the normal CAM that is not considered garbage. Moreover, the VAE, which has learned only the normal CAM, treats the garbage CAM input during the test process to be done later as an outlier.

## 5. Experiment and Performance Analysis

### 5.1. Simulations: Public Motor Dataset

To evaluate the performance of the proposed method, we used the Ford Motor Dataset, a public industrial dataset, in this paper. These data were originally used in a competition at the IEEE World Congress on Computational Intelligence in 2008 [33]. This is a dataset for classification of problems to detect and diagnose whether a fault exists in the automotive subsystem. It consists of two problems, FordA and FordB.

- FordA: Both training and test data were collected in a way that minimizes noise contamination under typical operating conditions.
- FordB: Training data were collected under typical operating conditions, but test data were collected under noisy conditions.

These data are suitable for experimenting with the proposed method because they include datasets that are difficult to diagnose when it is noisy and an industrial dataset for which diagnosis is relatively easy because it has low noise.

**Figure 2:**
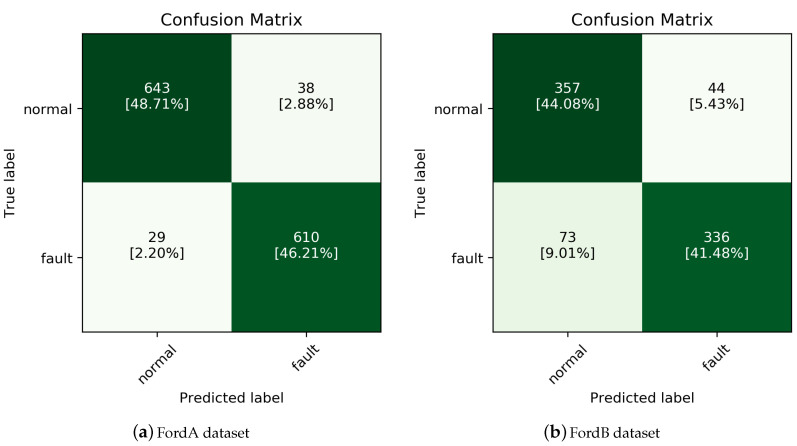
Fault detection result using inceptionTime [34] classifier.

Fault detection was done using the inceptionTime classifier [34] known to have the best performance in the Ford Motor Dataset; the result is depicted in Figure 2. The inceptionTime classifier is known as the state-of-the-art (SOTA) model for classifying time-series data collected in the form of a one-dimensional vector and consists of a 1D-CNN-based architecture. For Ford A and Ford B datasets, the model accuracy was 94.9% and 85.6%, respectively.

**Figure 3:**
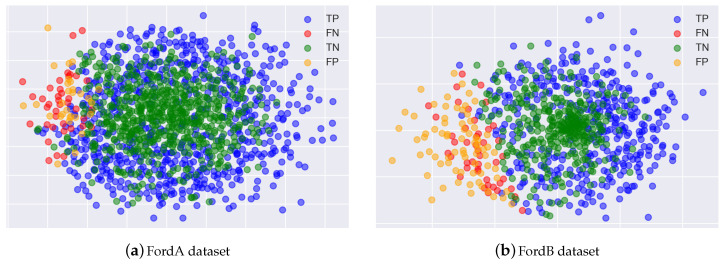
Result of visualization using MDS representation for garbage and normal CAM.

We assumed that normal CAM corresponding to TP and TN and garbage CAM corresponding to FP and FN would have different manifolds. To check this, we visualize after reducing the dimensions of the normal CAM and the garbage CAM using Multi-dimensional scaling (MDS), which locates structures or relationships between objects by placing them in a space of a dimension (usually two-dimensional) lower than the original dimension by using distance or dissimilarity between the objects [35]. Figure 3 shows the results of the visualization using MDS, which confirmed that the CAM generated with label information corresponding to FN and FP considered as garbage CAM can be separated almost linearly from the normal CAM.

Last, VAE training was done using only normal CAM to implement a latent variable-based control chart for proactively identifying garbage CAM that may provide misdiagnosis information to end-users. The α used to do the experiment was 1%. The outlier classification rule was set to the case where an alarm occurred in both the $T_{PCA}^{2}$ and the $Q_{PCA}$ charts. The number of principal components *p* was set to the point where the explained variance exceeds 90%.

Table 1 lists the results of applying the latent-variable-based control chart extracted from the VAE. After checking the CAM classified as outliers by means of the process-monitoring step, the newly calculated accuracy was increased for FordA to 99.5% and for FordB to 96.7%, based on the CAM that generated the true alarm and the data that caused the false alarm.

### 5.2. Case Study: Sapphire Nozzles Grinding Using MCT

To demonstrate the proposed method, we established a data collection and processing platform at the actual manufacturing site. We confirmed whether the proposed method could effectively improve fault detection and diagnostic performance. We conducted a case study at a manufacturer doing sapphire grinding at a machining center (MCT) using a diamond milling tool.

Sapphire is a crystalloid, single crystal that Al${}_{2}$O${}_{3}$ (Alumina) has grown into after becoming fluid with heat of over $2000{\;}^{\circ }C$ in the growth furnace [36]. It has a hardness and strength close to that of the diamond but also has high heat resistance. With this advantage, replacing parts made of ceramics, quartz, or silicon used in current semiconductors with sapphire material could reduce contaminants generated in chemical vapor deposition (CVD) and etching process chambers and improve yield.

However, because of the great hardness and strength of sapphire, there is a disadvantage in that the milling tool is prone to a fault in the grinding process using MCT. As a result, there is a high possibility of quality problems in products manufactured with milling tools in a fault condition. In order to improve the previous issue, we applied the proposed method to check whether the grinding milling tool of MCT is in normal condition or fault condition.

#### 5.2.1. Data Platform for Monitoring Streaming Process Data

**Figure 4:**
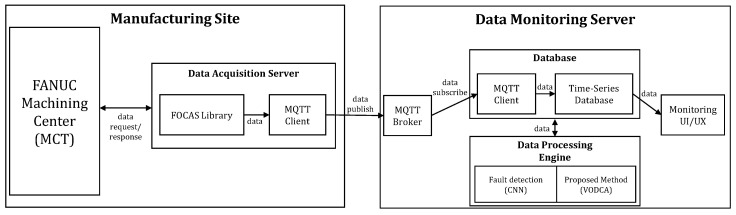
An overview of the data platform built to conduct case studies on the manufacturing site. Arrows indicate the direction in which data is streamed.

To apply the proposed method to the actual manufacturing site, we built a data platform that can do both data collection and processing. The overview of the data platform is shown in Figure 4.

We conducted a case study using an MCT equipped with a diamond milling tool for sapphire nozzle grinding. The specifications of the MCT on which we collected data for the experiment are shown in Table 2.

**Figure 5:**
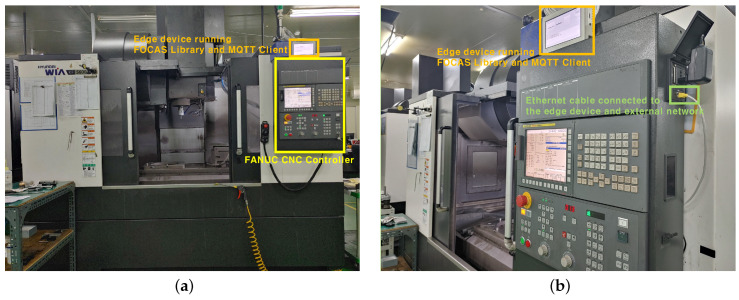
Connection status of MCT and edge device used in the case study. (**a**) MCT used in this case study. The edge device installed on the top of the CNC controller collects data through the FOCAS library and distributes the collected data through MQTT, (**b**) The edge device and the CNC controller are connected to an external network that allows access to the data monitoring server via an ethernet cable.

In order to collect data from the MCT, we used an edge device as a data collection server, and connected it to the MCT by means of an Ethernet cable, as shown in Figure 5 The edge device collects data using the FANUC FOCAS Library, which provides a reliable way to connect Fanuc FOCAS Ethernet controllers to OPC Client applications, including HMI, SCADA, Historian, MES, ERP, and countless custom applications. This Library is intended for use with the Fanuc FOCAS Computer Numerical Control (CNC) system [37].

Edge devices collects data by means of the FOCAS library and publishes the collected data to the monitoring server while also acting as a Message Queuing Telemetry Transport (MQTT) publisher. MQTT is one such machine-to-machine (M2M) connectivity protocol, recently standardized by OASIS. It was designed as a lightweight publish/subscribe messaging transport for small sensors and mobile devices. The publish-subscribe model is a messaging paradigm where the messages are published to a broker, and the receivers can subscribe to the broker to receive messages. The publishers and subscribers do not need to know each other because the broker is employed [38].

Process data transmitted to the data-monitoring server by means of MQTT are stored in the database. The stored data are processed by means of the proposed method in the data-processing engine. In this case study, to effectively process data streamed in real time, we used a time-series database instead of a commonly used relational database. The time-series database was built using Influxdb [39]. Relational databases can handle time-series data but are not optimized for typical time-series workloads. In contrast, the Time-series database is designed to store large amounts of time-series data and quickly do real-time analysis on such data.

#### 5.2.2. Experimental Setup for the Case Study

This section describes how we designed the experiment in the case study. As mentioned earlier, sapphire is prone to tool wear or breakage in grinding, because it is very hard and strong. This leads to product defects, resulting in the waste of raw material and tools and reduced yield. Even if tool breakage occurs, there are cases where the MCT does not set off an alarm and the idle time of the equipment increases.

**Figure 6:**
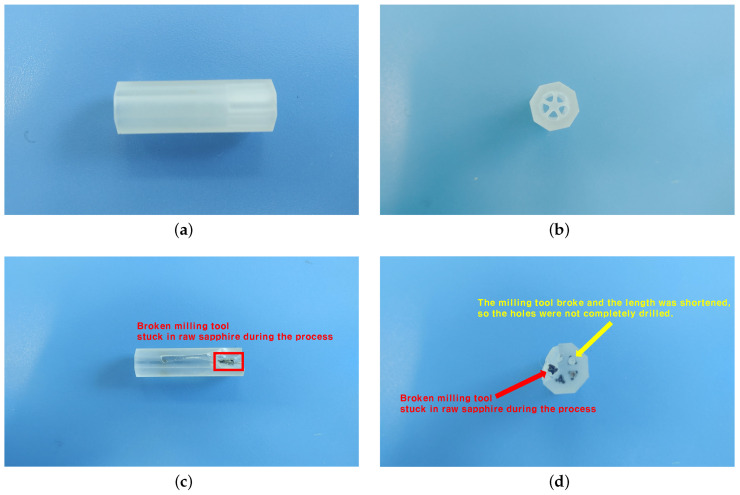
Samples of normal and defective sapphire nozzles depending on whether or not the milling tool is in a fault condition. (**a**) Normal Sapphire Nozzle (Side Angle Shot), (**b**) Normal Sapphire Nozzle (High Angle Shot), (**c**) Defective Sapphire Nozzle (Side Angle Shot), (**d**) Defective Sapphire Nozzle (High Angle Shot).

Figure 6 shows the most common product defects that occur in the manufacturing of sapphire nozzles by manufacturers who provided us with an experimental environment. These are defects in which a tool breaks while drilling a hole in the raw material, so that the broken milling tool gets stuck in the raw material. As the milling tool was stuck, the sapphire surface was cracked entirely. Also, since the milling tool breaks during the process, the shortened milling tool cannot wholly drill the hole.

In this case study, we defined the data collected when this type of fault occurs as fault data. For use in neural network learning, data such as the spindle motor’s load, the coordinate value of the milling tool, and current, which are directly related to the milling tool, were collected.

**Figure 7:**
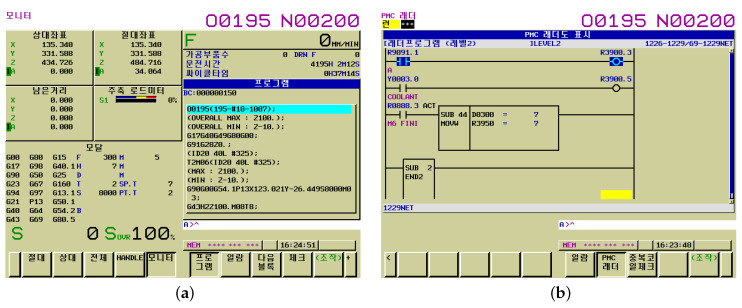
FANUC controller screen corresponding to the collected data. (**a**) The FANUC controller main screen, the coordinates of the milling tool, and the load are displayed, (**b**) FANUC controller Laddar screen, We set the number of the currently used milling tool at the PMC address corresponding to R3950.

The MCT works by automatically changing the various milling tools installed in the tool magazine, which is called auto tool change (ATC). The number of milling tools that can be mounted in the MCT’s tool magazine we used is 30ea, as shown in Table 2. We needed to keep track of which milling tools the data we were collecting came from. For this, a sequence program was created using FANUC LADDER-III. Through this, we set the number of the currently used milling tool at the programmable machine controller (PMC) address corresponding to R3950. The PMC is a sequence control unit that is built into the FANUC CNC controller. Figure 7 is the FANUC CNC controller screen corresponding to the collected data.

#### 5.2.3. Experimental Results of the Case Study

Data was collected at a sampling rate of 1 Hz. We preprocessed the collected data using a recurrence plot in order to effectively train a neural-network model for the instantaneous soaring load value. The recurrence plot (RP) is a method of transforming a one-dimensional vector into a two-dimensional matrix [40]. RP provides a way to visualize the periodic feature of a trajectory by means of a phase space and to investigate specific aspects of the n-dimensional phase-space trajectory by means of a 2D representation [41].

Accordingly, we used 2D-CNN-based neural-network architecture in the case study, unlike 1D-CNN in the previous section. The CNN model for processing data represented in 2D used MobileNetV2 [42] to speed up real-time data processing. In the case study, we used Grad-CAM instead of the CAM used in the previous simulation to explain the model’s detection results. The hyperparameter of the multivariate control chart for doing process monitoring is the same as in the previous experiment.

**Figure 8:**
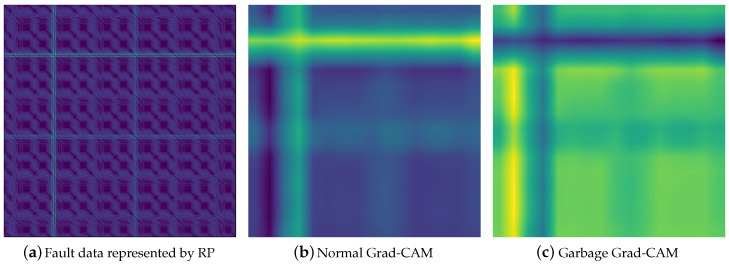
Grad-CAM generated differently in the success or failure of the CNN model’s fault detection.

Figure 8 shows the fault data represented in RP and Grad-CAM generated from the data. The high instantaneous load on the milling tool can be represented as a cross-shaped image by means of RP. When the CNN model’s fault detection is successful, the localization map generated by means of Grad-CAM highlights the part corresponding to the high load. However, if there is misclassification, the generated localization map does not highlight the part corresponding to the high load at all.

In a situation where the CNN model’s fault detection fails, and the misclassification occurs, if the diagnosis is not verified, secondary damage may occur because of the false diagnosis. In this case study, the accuracy of fault detection using MobileNetV2 was only 78.4%. However, when we did post-processing using the proposed method, the fault detection accuracy increased by about 8.8% to 87.2%.

## 6. Conclusions and Future Work

In this paper, we proposed a process-monitoring framework that can detect and diagnose faults occurring in the system based on GIGO logic. In the proposed framework, FDD is done using a CNN-based model. In the previous step, we trained a VAE that learns only the manifold of a normal CAM to verify the reliability of the CAM generated for diagnosis. By identifying the garbage CAM that may provide misdiagnosis information to the end user by means of a latent-variable-based control chart extracted by means of VAE, misdiagnosis information can be prevented from reaching the end user. The advantage of this is that it can achieve higher fault-detection accuracy than before, while providing end users with diagnosis results that have been verified for reliability, so that end users can trust the system.

As future work, studies that increase the generalizability of the proposed method by applying it to a wide range of industrial data are needed. In this study, we confirmed that manifolds of normal and garbage CAM could be distinguished. Accordingly, we applied process monitoring based on a multivariate control chart to separate normal and garbage CAM. In future work, we hope to research a technique to properly separate the garbage CAM using a boosting-based ensemble model.

## Figures and Tables

**Table 1:** Results of a latent variable-based control chart experiment (Simulation).

Metrics	FordA	FordB
Number of outliers in VAE	70	128
Number of garbage CAM	67	117
Number of true alarm	65	109
Number of Type 1 Errors	5	19
Number of Type 2 Errors	2	8
Type 1 Error Rate	7.1%	14.8%
Type 2 Error Rate	2.9%	6.8%
Accuracy:only CNN	94.9%	85.6%
Accuracy:with VAE	99.5%	96.7%
Increased Accuracy	4.6%	11.1%

**Table 2:** The specifications of the MCT we collected data to perform the experiment.

Equipment	Info
MCT	Hyundai Wia KF5600
Controller	FANUC Series 0i-MF
Spindle rotation speed	12000 RPM
Table size	1100 × 560 × 520 (H)
Rated voltage	AC 220V 3Ø
Rated frequency	60 Hz
Rated power capacity	28.5 KVA
Maximum allowable current	75 A
Rated breaking current	25 KA
Control voltage	AC 110, DC 24V
Milling tool holder	BT-40
Milling tool magazine	30ea

## References

[1] Ku W., Storer R.H., Georgakis C. (1995). Disturbance detection and isolation by dynamic principal component analysis. Chemom. Intell. Lab. Syst..

[2] Lee S., Kwak M., Tsui K.L., Kim S.B. (2019). Process monitoring using variational autoencoder for high-dimensional nonlinear processes. Eng. Appl. Artif. Intell..

[3] Domingos P. (2012). A few useful things to know about machine learning. Commun. ACM.

[4] Bengio Y., Courville A., Vincent P. (2013). Representation learning: A review and new perspectives. IEEE Trans. Pattern Anal. Mach. Intell..

[5] Wang J., Ma Y., Zhang L., Gao R.X., Wu D. (2018). Deep learning for smart manufacturing: Methods and applications. J. Manuf. Syst..

[6] Zhou B., Khosla A., Lapedriza A., Oliva A., Torralba A. Learning deep features for discriminative localization. Proceedings of the IEEE conference on computer vision and pattern recognition.

[7] Bahdanau D., Cho K., Bengio Y. (2014). Neural machine translation by jointly learning to align and translate. arXiv.

[8] Kim J., Kim J.M. (2020). Bearing Fault Diagnosis Using Grad-CAM and Acoustic Emission Signals. Appl. Sci..

[9] Xu K., Ba J., Kiros R., Cho K., Courville A., Salakhudinov R., Zemel R., Bengio Y. Show, attend and tell: Neural image caption generation with visual attention. Proceedings of the International conference on machine learning.

[10] Venkatasubramanian V., Rengaswamy R., Yin K., Kavuri S.N. (2003). A review of process fault detection and diagnosis: Part I: Quantitative model-based methods. Comput. Chem. Eng..

[11] Hwang I., Kim S., Kim Y., Seah C.E. (2009). A survey of fault detection, isolation, and reconfiguration methods. IEEE Trans. Control. Syst. Technol..

[12] Montgomery D.C. (2007). Introduction to Statistical Quality Control.

[13] Zhu X., Goldberg A.B. (2009). Introduction to semi-supervised learning. Synth. Lect. Artif. Intell. Mach. Learn..

[14] Zhao R., Yan R., Chen Z., Mao K., Wang P., Gao R.X. (2019). Deep learning and its applications to machine health monitoring. Mech. Syst. Signal Process..

[15] Gehrmann S., Strobelt H., Krüger R., Pfister H., Rush A.M. (2019). Visual Interaction with Deep Learning Models through Collaborative Semantic Inference. IEEE Trans. Vis. Comput. Graph..

[16] O’Shea T.J., Roy T., Erpek T. Spectral detection and localization of radio events with learned convolutional neural features. Proceedings of the 2017 25th European Signal Processing Conference (EUSIPCO).

[17] Selvaraju R.R., Cogswell M., Das A., Vedantam R., Parikh D., Batra D. Grad-cam: Visual explanations from deep networks via gradient-based localization. Proceedings of the IEEE International Conference on Computer Vision.

[18] Shewhart W.A. (1931). Economic Control of Quality of Manufactured Product.

[19] Page E.S. (1954). Continuous inspection schemes. Biometrika.

[20] Roberts S. (2000). Control chart tests based on geometric moving averages. Technometrics.

[21] Lowry C.A., Montgomery D.C. (1995). A review of multivariate control charts. IIE Trans..

[22] Hotelling H. (1947). Multivariate Quality Control. Techniques of Statistical Analysis.

[23] Seborg D.E., Mellichamp D.A., Edgar T.F., Doyle F.J. (2010). Process Dynamics and Control.

[24] Yu F., Qiu F., Meza J. (2016). Design and Statistical Analysis of Mass-Spectrometry-Based Quantitative Proteomics Data. Proteomic Profiling and Analytical Chemistry.

[25] Mastrangelo C.M., Runger G.C., Montgomery D.C. (1996). Statistical process monitoring with principal components. Qual. Reliab. Eng. Int..

[26] Box G.E. (1954). Some theorems on quadratic forms applied in the study of analysis of variance problems, I. Effect of inequality of variance in the one-way classification. Ann. Math. Stat..

[27] Van Sprang E.N., Ramaker H.J., Westerhuis J.A., Gurden S.P., Smilde A.K. (2002). Critical evaluation of approaches for on-line batch process monitoring. Chem. Eng. Sci..

[28] Schalkoff R.J. (2007). Pattern recognition. Wiley Encycl. Comput. Sci. Eng..

[29] Schölkopf B., Smola A., Müller K.R. (1998). Nonlinear component analysis as a kernel eigenvalue problem. Neural Comput..

[30] Ge Z., Yang C., Song Z. (2009). Improved kernel PCA-based monitoring approach for nonlinear processes. Chem. Eng. Sci..

[31] Mansouri M., Nounou M., Nounou H., Karim N. (2016). Kernel PCA-based GLRT for nonlinear fault detection of chemical processes. J. Loss Prev. Process. Ind..

[32] Malhotra P., Ramakrishnan A., Anand G., Vig L., Agarwal P., Shroff G. (2016). LSTM-based encoder-decoder for multi-sensor anomaly detection. arXiv.

[33] Abou-Nasr M., Feldkamp L. Ford Classification Challenge. http://www.timeseriesclassification.com/dataset.php.

[34] Fawaz H.I., Lucas B., Forestier G., Pelletier C., Schmidt D.F., Weber J., Webb G.I., Idoumghar L., Muller P.A., Petitjean F. (2019). InceptionTime: Finding AlexNet for Time Series Classification. arXiv.

[35] Kruskal J.B. (1964). Multidimensional scaling by optimizing goodness of fit to a nonmetric hypothesis. Psychometrika.

[36] Khattak C.P., Shetty R., Schwerdtfeger C.R., Ullal S. (2016). World’s largest sapphire for many applications. J. Cryst. Growth.

[37] Fanuc G. (2002). FOCAS1/2 Open CNC Libraries Documentation.

[38] Shinde S.A., Nimkar P.A., Singh S.P., Salpe V.D., Jadhav Y.R. (2016). MQTT-message queuing telemetry transport protocol. Int. J. Res..

[39] Naqvi S.N.Z., Yfantidou S., Zimányi E. (2017). Time series databases and influxdb. Stud. Univ. Libre Brux..

[40] Eckmann J., Kamphorst S.O., Ruelle D. (1995). Recurrence plots of dynamical systems. World Sci. Ser. Nonlinear Sci. Ser. A.

[41] Hatami N., Gavet Y., Debayle J. (2018). Classification of time-series images using deep convolutional neural networks. In Proceedings of the Tenth international conference on machine vision (ICMV 2017). Int. Soc. Opt. Photonics.

[42] Sandler M., Howard A., Zhu M., Zhmoginov A., Chen L.C. Mobilenetv2: Inverted residuals and linear bottlenecks. Proceedings of the IEEE conference on computer vision and pattern recognition.

